# Ring finger protein 126 (RNF126) suppresses ionizing radiation–induced p53-binding protein 1 (53BP1) focus formation

**DOI:** 10.1074/jbc.M116.765602

**Published:** 2017-11-22

**Authors:** Nam Soo Lee, Hae Ryung Chang, Soomi Kim, Jae-Hoon Ji, Joorak Lee, Hyun Ji Lee, Yoojeong Seo, Misun Kang, Joo Seok Han, Kyungjae Myung, Yonghwan Kim, Hongtae Kim

**Affiliations:** From the ‡ Department of Biological Sciences, Sungkyunkwan University, Suwon 16419, Republic of Korea,; the § Department of Biological Sciences, Sookmyung Women's University, Seoul 04310, Republic of Korea,; the ¶ Genomic Instability Research Center, Ajou University School of Medicine, Suwon 16499, Republic of Korea,; the ‖ Center for Genomic Integrity, Institute for Basic Science, Ulsan National Institute of Science and Technology, Ulsan 44919, Republic of Korea, and; the ** Center for Neuroscience Imaging Research, Institute for Basic Science, Sungkyunkwan University, Suwon 16419, Republic of Korea

**Keywords:** DNA damage, DNA damage response, histone modification, signal transduction, ubiquitylation (ubiquitination)

## Abstract

Cells have evolved sophisticated mechanisms to maintain genomic integrity in response to DNA damage. Ionizing radiation (IR)–induced DNA damage results in the formation of IR-induced foci (iRIF) in the nucleus. The iRIF formation is part of the DNA damage response (DDR), which is an essential signaling cascade that must be strictly regulated because either the loss of or an augmented DDR leads to loss of genome integrity. Accordingly, negative regulation of the DDR is as critical as its activation. In this study, we have identified ring finger protein 126 (RNF126) as a negative regulator of the DDR from a screen of iRIF containing 53BP1. RNF126 overexpression abolishes not only the formation of 53BP1 iRIF but also of RNF168, FK2, RAP80, and BRCA1. However, the iRIF formation of γH2AX, MDC1, and RNF8 is maintained, indicating that RNF126 acts between RNF8 and RNF168 during the DDR. In addition, RNF126 overexpression consistently results in the loss of RNF168-mediated H2A monoubiquitination at lysine 13/15 and inhibition of the non-homologous end joining capability. Taken together, our findings reveal that RNF126 is a novel factor involved in the negative regulation of DDR, which is important for sustaining genomic integrity.

## Introduction

Cells have evolved sophisticated mechanisms to maintain genomic integrity in response to both exogenous and endogenous DNA damage (1). DNA damage induced by ionizing radiation (IR)^4^ leads to the formation of distinctive foci in the nucleus that are referred to as IR-induced foci (iRIF). In the event of iRIF, DNA damage response (DDR) factors are recruited to the sites of DNA damage. These factors retain genomic integrity by regulating cell cycle checkpoints (1) and DNA damage repair (2). Histone phosphorylation initiates the DNA damage repair pathway and serves as a marker of sites of DNA damage. Histone phosphorylation also later serves as a platform for assembly of downstream DDR factors, such as RAP80/BRCA1 and 53BP1 (3). These factors localize to γH2AX, a variant of H2A phosphorylated by PI3K-like kinases, including ataxia telangiectasia-mutated (ATM), ATM and Rad3-related (ATR), and DNA-dependent PKs (DNA-PKs) (4, 5). Initially, γH2AX recruits mediator of DNA damage checkpoint protein 1 (MDC1), which interacts via its tandem BRCA1 C terminus domain (BRCT) (6). RNF8 then localizes to sites of DNA damage through direct interaction with phosphorylated MDC1 and ubiquitinates linker histone H1 (7). This ubiquitination then attracts motif interacting with ubiquitin of RNF168 (8–11). Together with the E2 ubiquitin-conjugating enzyme UBC13, RNF8 and RNF168 coordinate monoubiquitination and Lys-63–linked polyubiquitination of either H2A or H2AX. This process leads to incremental signals for DNA damage repair (8–10, 12, 13). Consequently, the ubiquitination of histones results in the recruitment of DNA repair machinery for homologous recombination and non-homologous end joining (NHEJ) (14–17).

Negative regulation of the DDR is as important as activating DNA damage repair pathways and is crucial for sustaining genomic integrity. The deubiquitination of histones is a negative regulatory mechanism of the DDR. BRCA1/BRCA2-containing complex subunit 36 (BRCC36), a component of the BRCA1 protein complex, deubiquitinates the Lys-63–linked polyubiquitination chains generated by RNF8 and RNF168 at the sites of DNA double-strand breaks (DSBs) (18). The ubiquitin-specific proteases (USP) 3 and 44, which promote deubiquitination of H2A/H2AX and thus prevent 53BP1 and BRCA1 iRIF formation, are additional negative regulators of the DDR (19, 20). In addition to USP 3 and 44, the ovarian tumor deubiquitinase ubiquitin aldehyde binding 1 (OTUB1) interferes with the interaction between RNF168 and UBC13 and inhibits H2A ubiquitination (21). Two homologous to the E6-AP carboxyl terminus (HECT) domain E3 ligases, TRIP12 and UBR5, regulate RNF168 expression levels and protein loading onto chromatin and suppress further ubiquitination (22). However, the relationship between the negative regulators has not yet been discovered.

Here we report the novel negative regulator RNF126 that was identified through a screen examining 53BP1 focus formation with individual expression of a group of proteins harboring the really interesting new gene (RING) domain. We found that RNF126 overexpression abolishes 53BP1 and BRCA1 iRIF formation and consistently inhibits NHEJ. Systematic immunofluorescence experiments involving iRIF formation of the DDR factors revealed that RNF126 acts between RNF8 and RNF168 and leads to the loss of H2A ubiquitination at Lys-13 and Lys-15. Taken together, our findings implicate that RNF126, as a negative regulator of the DDR, functions to maintain genomic stability.

## Results

### Identification of RNF126 as a novel negative regulator of IR-induced 53BP1 focus formation

**Figure 1.**
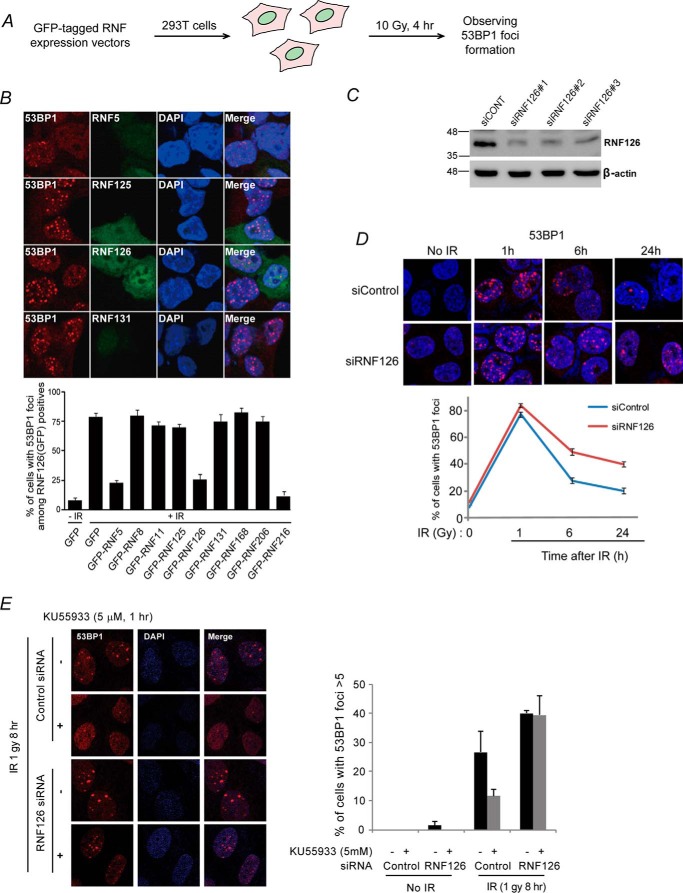
**Identification of a negative regulator of 53BP1 foci formation.** *A*, schematic of the screen used in this study. *B*, the indicated RNF protein expression vectors were transfected into 293T cells. After 48 h, the transfected 293T cells were exposed to 10 Gy of ionizing radiation. Four hours after the irradiation, the cells were fixed and stained with anti-53BP1 antibody. DAPI was used as a nuclear indicator. The results represent the average of two independent experiments. *Error bars* indicate the standard deviation. *C*, Knockdown of RNF126 by transfection with siRNA against RNF126. Control or RNF126 siRNA #1-, #2-, or #3-transfected cell lysates were analyzed by SDS-PAGE and immunoblotting with the specified antibodies. *D*, 293T cells were transfected with control or RNF126 siRNA #1. After 48 h, transfected 293T cells were exposed to 2 Gy of ionizing radiation. After 0, 1, 6, or 24 h, cells were fixed and stained with anti-53BP1 antibody. *E*, U2OS cells were transfected with control or siRNF and then treated with the ATM inhibitor KU55933 for 1 h at 5 μm. Cells were then irradiated at 1 Gy. Immunofluorescence detection of 53BP1 foci was observed 8 h after irradiation, and statistics was analyzed.

Various factors that promote or repress ubiquitination at sites of DNA damage play key roles balancing the DNA damage response. To identify a RING finger protein that negatively regulates the DDR and DNA repair pathways, we generated an inventory of expression vectors for functionally uncharacterized RING finger proteins harboring a conserved RING motif. Given that we tagged each of the RING finger proteins with GFP, the transfected cells were easy to distinguish from non-transfected cells. 293T cells expressing an individual RING finger protein were irradiated. Next we screened proteins suppressing IR-induced 53BP1 focus formation, a marker of DNA damage (17) (Fig. 1*A*). As shown in Fig. 1*B*, IR-induced 53BP1 focus formation was suppressed in cells expressing RNF5, RNF126, or RNF216. Of these proteins, we decided to further characterize RNF126 because it was predicted to interact with OTUB1, a known negative regulator of the DDR (23). The DDR-suppressing function of RNF126 was further supported by an observation of persistent 53BP1 iRIF formation in cells with RNF126 depletion (Fig. 1, *C* and *D*). 53BP1 foci appeared after 1 h of IR in both siControl and siRNF126-transfected cells, but the foci persisted after 24 h only in the siRNF126-transfected cells (Fig. 1*D*), implying that depletion of RNF126 results in enhanced IR-induced 53BP1 focus formation. It is worth to note that the persistent IR-induced 53BP1 focus formation might be due to the loss of factors that positively regulate DNA damage responses. To gain more insights into the functional roles of RNF126 in the DNA damage response, we performed additional experiments. Nakada *et al.* (21) revealed that OTUB1 negatively regulates the DDR by inhibiting the RNF168 pathway and showed that depletion of the negative DDR regulator is able to partially rescue the suppressed IR-induced 53BP1 focus formation caused by ATM inhibitor. These results can be interpreted as follows. Although OTUB1 functions downstream of ATM in response to DNA damage, because pharmaceutical inhibition of the ATM is partial, removal of the DDR negative regulator OTUB1 would increase the DNA damage response. To further investigate the role of RNF126 in the DDR, the ATM inhibitor KU55933 was applied to cells transfected with control or RNF126 siRNA to evaluate whether a similar result is observed as when OTUB1 is depleted. To this end, IR-induced 53BP1 foci were observed using immunofluorescence. As expected, the number of IR-induced 53BP1 foci drastically decreased with KU55933 treatment of control cells, which was successfully rescued by depletion of RNF126, further suggesting negative regulator roles of RNF126 (Fig. 1*E*). In addition, we performed a clonogenic assay to determine the function of RNF126 in response to DNA damage induced by IR and found that overexpression of RNF126 leads to reduced cell survival, implying that impairment of the DDR because of overexpression of RNF126 results in reduced cell survival in response to IR (Fig. S1, *A–C*). Taken together, these findings suggest that RNF126 might function as a negative regulator of IR-induced 53BP1 focus formation.

**Figure 2.**
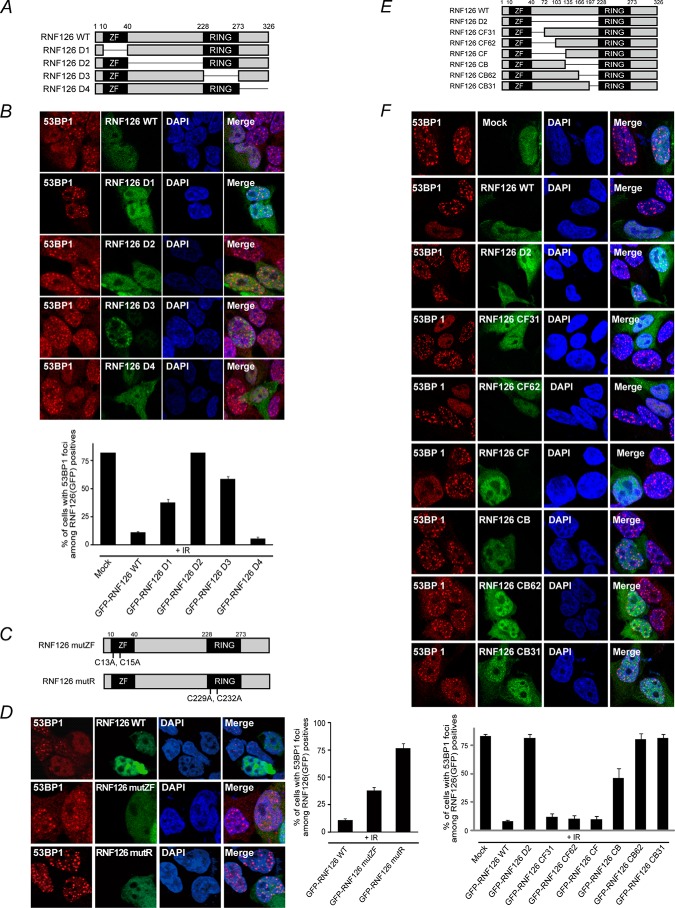
**Identification of RNF126 regions to inhibit 53BP1 focus formation.** *A*, *C*, and *E*, diagrams of WT RNF126, serial deletion mutants, and point mutation constructs. *B*, *D*, and *F*, RNF126 inhibits 53BP1 focus formation through its ZF, central region, and RING domains. 293T cells were transfected with plasmids encoding WT RNF126 and serial deletion mutants. After 48 h, the transfected 293T cells were exposed to 10 Gy of ionizing radiation. Four hours after irradiation, the cells were fixed and stained with anti-53BP1 antibody. The results represent the average of two independent experiments. *Error bars* indicate the standard deviation for each expression plasmid–transfected cell.

RNF126 consists of 326 amino acids and contains both RING and zinc finger (ZF) domains, both of which are highly conserved between species (Fig. S2). To determine the critical domains of RNF126 that are responsible for its inhibitory activity on 53BP1 iRIF formation, we generated a series of internal deletion mutants of GFP-tagged RNF126 (RNF126-D1 to RNF126-D4) (Fig. 2*A*) and transfected each of the RNF126 deletion mutants into 293T cells. After irradiating the cells, endogenous 53BP1 focus formation was detected by immunofluorescence. As shown in Fig. 2*B*, the expression of the RNF126 WT and RNF126-D4 mutants effectively suppressed 53BP1 iRIF formation. However, RNF126-D1, D2, and D3 failed to suppress 53BP1 iRIF formation, indicating that the RING, central region, and ZF motifs of RNF126 are important for the negative regulatory functions of RNF126. Within the two well-established RING and ZF domains, we mutated highly conserved amino acid residues that are critical for its function. Cys-13 and Cys-15 within the ZF domain (RNF126 mutZF) and Cys-229 and Cys-232 in the RING domain (RNF126 mutR) were substituted with alanine. Expression of these point mutations in 293T cells further confirmed that the RING and ZF motifs of RNF126 are important for the negative regulatory function of RNF126 (Fig. 2, *C* and *D*). Interestingly, despite no known identified annotated motifs, the central region of RNF126 appears to be the most important for the function of RNF126 in suppressing 53BP1 iRIF formation. To narrow down the region responsible for the suppression, the central region was further divided into six regions (Fig. 2*E*). Transfection of each of these mutants revealed that RNF126-CB31, CB62, and CB failed to suppress 53BP1 iRIF formation (Fig. 2*F*).

Factors implicated in the DDR are supposed to be inactive when there is no DNA damage. They should be activated in response to DNA damage only because an inappropriate DDR causes unnecessary cell cycle arrest or even cell death. The monomer–dimer transition is one mechanism that modulates protein function. Indeed, functionally active forms of some of the DDR factors, including ATM, CHK2, and MDC1, are determined at the monomer–dimer transition (24–26). Furthermore, RING finger proteins can function as monomers, dimers, or multisubunit complexes. These findings prompted us to assess whether RNF126 forms a dimer. To demonstrate the dimerization of RNF126, we generated HA- and GFP-tagged RNF126 expression plasmids (HA-RNF126 and GFP-RNF126, respectively). Then we performed an immunoprecipitation assay with the cell lysates transfected with HA-RNF126 and GFP-RNF126 expression vectors. GFP-RNF126 co-immunoprecipitates HA-RNF126, and HA-RNF126 consistently co-immunoprecipitates GFP-RNF126 (Fig. S3, *A* and *B*), indicating that RNF126 forms a dimer. To determine the domain responsible for RNF126 dimerization, immunoprecipitation was performed using HA-RNF126 and a series of GFP-RNF126 deletion mutants (described in Fig. 2, *A* and *E*). Interestingly, we found that the region responsible for RNF126 homodimerization is the central region of RNF126, which is also critical for inhibiting IR-induced 53BP1 focus formation (Fig. S3, *C* and *D*). Dimerization of the RNF126-CB deletion mutant was compromised, similar to that of the D2 deletion mutant, but shorter deletion of this region, CB31 and CB62, still retained partial dimerization ability, although CB62 seems to be slightly less effective. Because the RNF126-CB mutant failed to inhibit 53BP1 iRIF and RNF126 homodimerization, it is possible that homodimerization of RNF126 is important for 53BP1 iRIF formation. However, we cannot exclude the possibility of another mechanism required for the inhibitory function of RNF126, as CB31 and CB62 deletion mutants form 53BP1 foci, although they are still able to form a dimer.

### RNF126 acts between RNF8 and RNF168 during IR-induced 53BP1 focus formation

**Figure 3.**
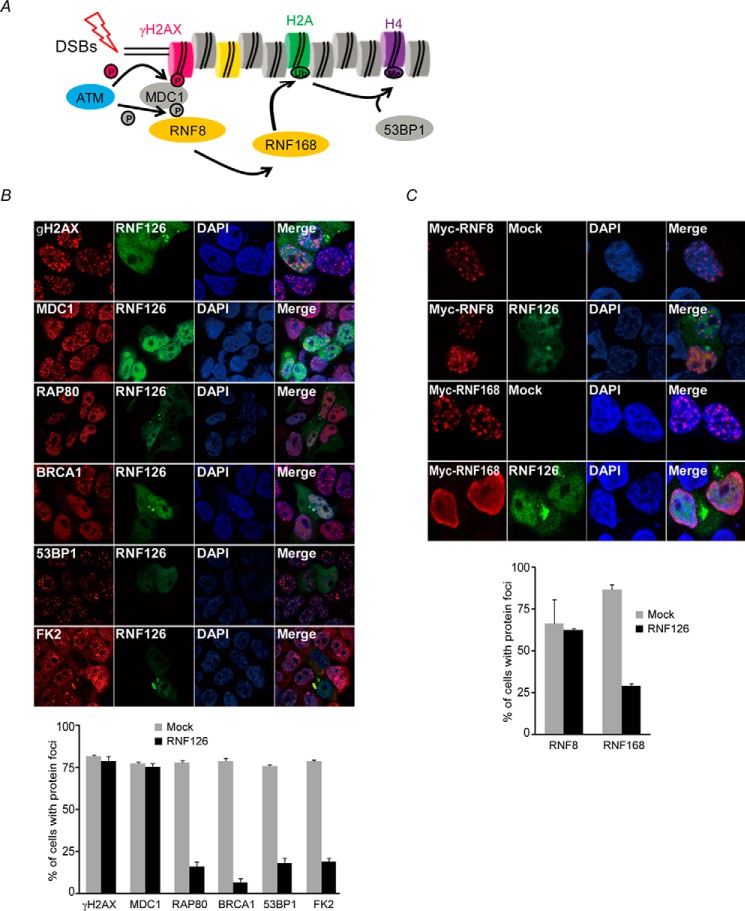
**RNF126 regulates recruitment of repair proteins to DSBs.** *A*, schematic of the IR-induced DNA damage signaling cascade. *B*, 293T cells were transfected with the GFP-RNF126 expression plasmid. After 48 h, the transfected 293T cells were exposed to 10 Gy of ionizing radiation. Four hours after irradiation, the cells were fixed and stained with an anti-γH2AX, -MDC1, anti-FK2, anti-RAP80, anti-BRCA1, or anti-53BP1 antibody. DAPI was used as a nuclear indicator. The results represent the average of two independent experiments. *Error bars* indicate the standard deviation for each expression plasmid–transfected cell. *C*, Myc-RNF8 or Myc-RNF168 with/without the GFP-RNF126 plasmid was transfected into 293T cells. After 48 h, transfected 293T cells were exposed to 10 Gy of ionizing radiation. Four hours after irradiation, the cells were fixed and stained with an anti-Myc antibody. DAPI was used to indicate the nuclei. The results represent the average of two independent experiments. *Error bars* indicate the standard deviation for each expression plasmid–transfected cell.

The hierarchy of RNF8- and RNF168-mediated ubiquitination-dependent IR-induced 53BP1 focus formation has been well characterized. After DNA damage, ATM and its related kinase phosphorylate H2AX. γH2AX is then recognized by MDC1, which provides a docking platform for downstream DDR factors such as RNF8, RNF168, and 53BP1 sequentially (Fig. 3*A*). To elucidate the point at which this hierarchy of RNF126 functions to suppress 53BP1 focus formation after DNA damage, we analyzed γH2AX, MDC1, RNF8, ubiquitin conjugation on chromatin (detected by FK2 antibody), RNF168, 53BP1, RAP80, and BRCA1 iRIF formation in cells expressing GFP-tagged RNF126. As clearly shown in Fig. 3, *B* and *C*, γH2AX, MDC1, and RNF8 iRIF formation in cells expressing GFP-RNF126 was comparable with cells with mock transfection. By contrast, RNF168, 53BP1, RAP80, and BRCA1 iRIF formation as well as ubiquitin conjugation on chromatin were severely impaired. Taken together, these findings suggest that the position of RNF126-dependent inhibition during IR-induced 53BP1 focus formation is downstream of RNF8 and upstream of RNF168.

### RNF126 inhibits RNF168-mediated H2A ubiquitination

**Figure 4.**
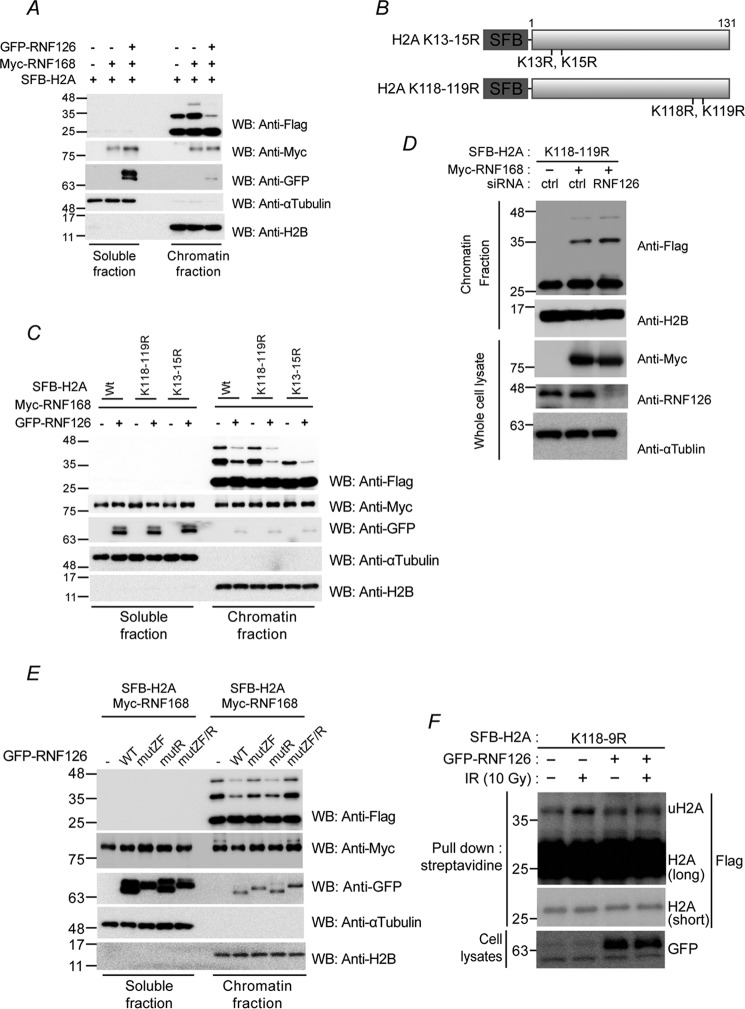
**RNF126 regulates H2A monoubiquitination.** *A*, *C*, and *E*, 293T cells were transfected with the indicated expression vectors. After 48 h, the transfected 293T cells were fractionated into soluble and chromatin fractions and then subjected to immunoblot analysis with the indicated antibodies. *WB*, Western blot. *B*, diagram of SFB-H2A point mutants. *D*, 293T cells were transfected with a combination of siRNA and expression vectors. After 48 h, the transfected 293T cells were fractionated into soluble and chromatin fractions and then subjected to immunoblot analysis with the indicated antibodies. *ctrl*, control. *F*, 293T cells were transfected with the indicated expression vectors. After 48 h, transfected 293T cells were exposed to 10 Gy of ionizing radiation. One hour after irradiation, the cell lysates were pulled down with streptavidin beads and then subjected to the indicated antibodies.

RNF168-mediated H2A ubiquitination is critical for the translocation of 53BP1 and RAP80 to sites of DNA damage (13). Because we demonstrated previously that expression of RNF126 suppresses ubiquitin conjugation on chromatin detected by an FK2 antibody, we assessed whether RNF126 inhibits RNF168-mediated H2A ubiquitination. Thus, we expressed SFB-tagged (S-tag, FLAG epitope tag, and streptavidin-binding peptide tag) H2A together with Myc-tagged RNF168 in the presence or absence of RNF126. H2A ubiquitination was determined using Western blot analysis of the chromatin fraction, and the eluates of H2A immunoprecipitates and the migration of ubiquitinated H2A were observed at 35 kDa (Fig. 4*A*). H2A ubiquitination increased in 293T cells expressing RNF168 (Fig. 4*A*, compare the *fourth* and *fifth lanes*), which is impaired by additive expression of RNF126 (Fig. 4*A*, compare the *fifth* and *sixth lanes*), implying that RNF126 suppresses RNF168-mediated H2A ubiquitination. H2A ubiquitination can occur at either Lys-118/Lys-119, markers for transcriptional repression, or Lys-13/Lys-15, which are tightly associated with a DNA DSB signaling cascade. To determine the sites of RNF126-dependent suppression of ubiquitination, we generated K118R and K119R SFB-H2A (SFB-H2A-K118–9R) and K13R and K15R SFB-H2A (SFB-H2A-K13–5R) expression vectors (Fig. 4*B*). As expected, RNF126-dependent suppression is associated with H2A ubiquitination, as seen by a decrease in ubiquitinated H2A intensity in cells with RNF126 overexpression (Fig. 4*C*). Because Lys-13 and Lys-15 ubiquitination is associated with RNF168 and the DDR, we further evaluated whether knockdown of RNF126 had any effect. Consistently, RNF126 depletion resulted in a slight increase in RNF168-dependent H2A ubiquitination at Lys-13 and Lys-15 (Fig. 4*D*). Next we investigated the domains responsible for RNF126 inhibition functions on H2A ubiquitination. Because we previously demonstrated that ZF and RING motifs as well as a central region are important for inhibiting 53BP1 focus formation, we assessed the effects of RNF126 on H2A monoubiquitination using ZF and/or RING domain point mutants. ZF or RING point mutants inhibited ∼50% of H2A mono-ubiquitination. ZF/RING double point mutants failed to inhibit H2A monoubiquitination (Fig. 4*E*), indicating that both the ZF and RING domains are responsible for inhibiting H2A monoubiquitination. Consistently, IR-induced H2A monoubiquitination was also inhibited by RNF126 (Fig. 4*F*).

### RNF126 translocation to DNA damage sites is dependent on the ZF domain

**Figure 5.**
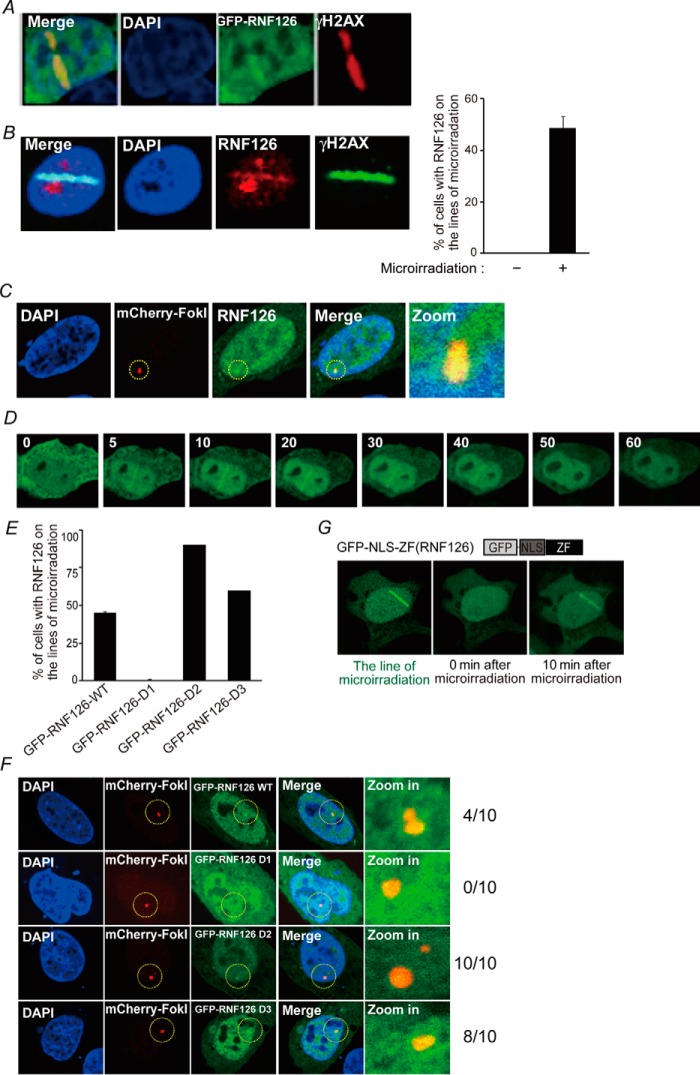
**Subcellular localization of RNF126 in response to DNA damage.** *A*, U2OS cells were transfected with GFP-RNF126 fusion protein expression vectors, and after 48 h, cells were treated with laser microirradiation. After 10 min, the cells were fixed and stained with anti-γH2AX. DAPI was used to indicate the nuclei. *B*, U2OS cells were treated with laser microirradiation. The cells were fixed and stained with anti-RNF126 and -γH2AX. The results represent the average of three independent experiments. *Error bars* indicate the standard deviation. *C*, mCherry-LacI-FokI was transfected with the indicated expression vectors into U2OS-DSB reporter cells. After 48 h, live-cell imaging was performed by confocal microscopy. *D*, the kinetics of GFP-RNF126 translocation to sites of DNA damage. *E* and *F*, GFP-RNF126 translocation to DNA damage sites is dependent on its ZF domain. The results represent the average of three independent experiments. *Error bars* indicate the standard deviation. *G*, GFP-RNF126 NLS-ZF translocation to sites of DNA damage.

Because RNF126 inhibits H2A monoubiquitination, we hypothesized that RNF126 may translocate to DNA damage sites after DNA damage. To assess this hypothesis, we used laser microirradiation and an mCherry-LacI-Fok I nuclease fusion protein to create a single DSB. As shown in Fig. 5*A*, upon microirradiation, GFP-RNF126 accumulated at the sites of DNA damage (50% in U2OS cells), and colocalized with γH2AX, a marker of DNA damage. Using an RNF126-specific antibody, we also confirmed that endogenous RNF126 accumulates at sites of laser-induced DNA lesions (Fig. 5*B*). These findings were supported by the different experimental sets, demonstrating that WT RNF126 colocalized with the mCherry-Fok I nuclease-induced single DSB site (Fig. 5*C*). GFP-RNF126 rapidly translocated to sites of DNA damage within 10 min after laser microirradiation. The translocation peaked at 20 or 30 min and gradually declined at 1 h (Fig. 5*D*). We next determined the specific regions of RNF126 responsible for its translocation to DNA damage sites. RNF126 WT as well as the D2, D3, and D4 mutants localized to the laser strip and at the single DSB site generated by mCherry-Fok I nuclease, whereas RNF126-D1 lacking the ZF domain did not localize to these sites (Fig. 5, *E* and *F*). This finding indicated that the ZF region is critical for the translocation to DNA damage sites. Interestingly, RNF126-D2 translocation efficiency is increased compared with RNF126 WT; however, the molecular basis of the observation remains elusive. It is possible that the loss of homodimerization increases the localization efficiency of RNF126 to the DSB site (Fig. S3*C*). To exclude the possibility that an altered protein conformational change was responsible for the inability of the RNF126-D1 mutant to localize to DNA damage sites, we generated an expression vector that exclusively expressed the ZF region of RNF126 (GFP-NLS-ZF, amino acids 11–40). Cells expressing GFP-NLS-ZF were subjected to laser microirradiation. We found that the ZF region of RNF126 is sufficient for localization to the laser strip (Fig. 5*G* and Fig. S4).

Recent studies revealed that some ZF domains are capable of binding to ubiquitin and have been renamed ubiquitin-binding ZNF domains (27). We examined whether the ZF domain of RNF126 would bind to ubiquitin *in vitro*. Using a ubiquitin–GST fusion protein (Ubi-GST), we demonstrated that Ubi-GST specifically binds to RNF126 WT but not to a RNF126 mutant that lacks the ZF domain (RNF126-D1) or contains a ZF point mutant (RNF126 mutZF) (Fig. S5, *A* and *B*). Additionally, the RNF126 ZF domain strongly binds to polyubiquitin (Fig. S5*C*). This ubiquitin-binding activity of the RNF126 ZF domain *in vitro* is consistent with its ability to localize to damage-induced foci *in vivo*, suggesting that RNF126 potentially associates with certain RNF8/UBC13-catalyzed ubiquitinated protein(s) at DSBs.

### RNF126 expression negatively regulates non-homologous end joining

**Figure 6.**
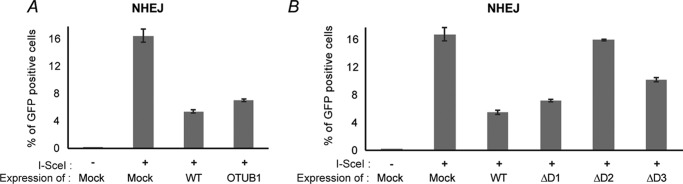
**RNF126 is required for DNA damage repair.** *A* and *B*, U2OS cells harboring the NHEJ reporter system were transfected with the indicated expression vectors. After 24 h, the cells were transfected again together with the I-SceI expression vector. After 72 h of incubation, the GFP expression level was analyzed by flow cytometry.

So far, we have provided evidence supporting that RNF126 expression not only abolishes IR-induced 53BP1 focus formation but also suppresses ubiquitin conjugation at Lys-13 and Lys-15 in H2A. These findings raise the possibility that RNF126 impairs RNF168-mediated ubiquitination at sites of DNA damage. This event consequently negatively regulates NHEJ. To assess the cytological effects of RNF126 expression on NHEJ, we employed U2OS reporter cell lines for NHEJ. The cells stably express the NHEJ reporter system, where, upon expression of the endonuclease I-SceI, GFP is expressed when NHEJ occurs properly. In this experiment, as a negative control, we overexpressed OTUB1, a known negative regulator of the DDR (21). As expected, RNF126 WT expression severely represses NHEJ frequency at levels similar to OTUB1 expression (Fig. 6*A*). Next we investigated the inhibition effects on NHEJ using the RNF126 deletion mutants to determine which domains are critical for NHEJ. Consistent with our previous results, expression of RNF126-D2 failed to suppress NHEJ, demonstrating that RNF126 homodimerization is critical for the negative regulatory function of RNF126 in the DDR (Fig. 6*B* and Fig. S6).

## Discussion

In this study, we identified and characterized RNF126 as a novel negative regulator of RNF168-mediated ubiquitination and 53BP1 focus formation after DNA damage. Previous studies revealed the role of RNF126 as a positive regulator of DSB repair by controlling the expression level of BRCA1 in homologous recombination and by its ubiquitin ligase function in NHEJ (28, 29). These reports highlighted the positive regulation of RNF126 in DSB repair, and our study elucidates a novel regulatory function of RNF126 in the DDR. We observed prolonged 53BP1 foci when RNF126 was knocked down, which poses two possibilities: first, that DNA double-strand break repair has been inhibited by knockdown of RNF126, implying that RNF126 is necessary for DNA damage repair, or second, that normally RNF126 plays a regulatory role in the DNA damage response and that, in the case of knockdown of RNF126, the DDR is enhanced. Because the DDR has multiple pathways involved, there are several genome maintenance regulatory factors that play both positive and negative roles depending on the stage, or these pathways regulate each other (1, 2). The first scenario concerning the role of RNF126 in DNA repair has been reported previously (28, 29). In this study, we focused on the DNA damage response steps prior to repair.

Based on the experimental data described above, we propose that the molecular basis of the inhibitory functions of RNF126 may be through inhibition of RNF168-mediated ubiquitination of H2A. Overexpression of RNF126 decreases H2A ubiquitination predominantly at Lys-13 and Lys-15, and the ZF and RING domains are responsible for the ubiquitination activity. On the other hand, RNF126 is able to form a homodimer, which is dependent on the D2 domain. This domain is also significant in 53BP1 focus formation, as the overexpression of WT RNF126 suppresses iRIF formation, but the D2 deletion mutant is unable to suppress it. Although the D2 domain is significant for both dimerization and iRIF formation, the exact location of each function may be different. The CB31 deletion mutant fails to suppress 53BP1 foci but is still capable of dimerization, which implies that these two functions are dependent on slightly different regions.

Amino acid sequences of RNF126 are highly conserved among species, which emphasizes the important physiological roles of RNF126 in cells. Initially, RNF126 was identified as an E3 ligase involved in ubiquitin-dependent protein degradation that targeted factors including p21 and activation-induced cytidine deaminase. However, the novel roles of RNF126 in the DDR are rarely affected by the RING motif. It was reported that Otub1, a deubiquitinase, also negatively regulates the DDR in a catalytically independent manner by regulating the activity of UBC13, an E2 ubiquitin-conjugating enzyme for RNF8 (21). Therefore, it would be interesting to study the regulatory mechanism of functional activity of RNF8 as a central point for fine-tuning of DDRs, which should be carefully managed because unnecessary activation of the DDR leads to cell cycle arrest and cell death.

## Experimental procedures

### Plasmids

GFP-tagged RNF protein expression plasmids were cloned into a GFP-tagged mammalian expression vector. GFP-tagged RNF126 point or serial deletion mutant expression plasmids were cloned into a GFP-tagged mammalian expression vector. A Myc-tagged RNF8 or RNF168 expression plasmid was cloned into a Myc-tagged mammalian expression vector. The SFB-tagged H2A or point mutant expression plasmids were cloned into a SFB-tagged mammalian expression vector. The SFB-tagged RNF8 or serial deletion mutant expression plasmids were cloned into an SFB-tagged mammalian expression vector.

### Cell culture

The U2OS, HeLa, and HEK 293T cell lines were purchased from the ATCC (Manassas, VA). The U2OS, HeLa, and 293T cell lines were maintained in DMEM (Invitrogen) supplemented with 10% FBS (Gibco) and 1% penicillin/streptomycin (Gibco) at 37 °C in 5% v/v CO_2_.

### siRNAs

The control siRNA used in this study was described previously (16, 30). The sequences of RNF126 siRNA were as follows: RNF126 #1, 5′ GCA GCA GGA UGA GAC CAA AUU 3′; RNF126 #2, 5′ GCA AGU UGC AGA CAG UCU AUU 3′. siRNAs were transfected into cells using Lipofectamine RNAiMAX reagent (Invitrogen).

### Antibodies

The properties of anti-RAP80, anti-MDC1, anti-BRCA1, anti-53BP1, and anti-γH2AX antibodies have been described previously (16, 30). Anti-FLAG, anti-β-actin, and anti-tubulin antibodies were purchased from Sigma. Myc and HA antibodies were purchased from Roche. RNF126 and RNF8 antibodies were purchased from Abcam.

### Transfection and immunoprecipitation

A transient transfection was performed using polyethyleneimine. Immunoprecipitation was done in two different protocols. Histone IP (streptavidin pulldown) was modified from a previous method (13), and transiently transfected 293T cells were harvested directly after irradiation. After a PBS wash, cells were lysed and sonicated in NETN (0.5% Nonidet P-40, 20 mm Tris [pH 8.0], 50 mm NaCl, 50 mm NaF, 100 μm Na_3_VO_4_, 1 mm DTT, and 50 μg/ml PMSF) at 4 °C. Crude lysates were cleared by centrifugation at 14,000 rpm at 4 °C for 5 min. Supernatants were incubated with streptavidin beads. Immunocomplexes were washed three times with NETN buffer and subjected to SDS-PAGE. For RNF126 homodimerization IP, 293T cells were washed with ice-cold PBS and then lysed in NETN buffer at 4 °C for 10 min. Crude lysates were cleared by centrifugation at 14,000 rpm at 4 °C for 5 min. Supernatants were incubated with protein A–agarose–conjugated primary antibodies. Immunocomplexes were washed three times with NETN buffer and subjected to SDS-PAGE. Western blotting was performed using the antibodies indicated in each figure legend.

### Chromatin fraction assay

293T cells were transfected with the designated plasmids. After 48 h, cells were harvested and washed once with PBS. Cell pellets were resuspended in NETN buffer and incubated on ice for 20 min. Lysates were centrifuged at 14,000 rpm at 4 °C for 10 min. The supernatant was the soluble fraction, which contained cytoplasmic proteins, and the pellets, which had the nuclei, were resuspended in 0.2 n HCl and incubated for 20 min on ice. Lysates were centrifuged at 14,000 rpm at 4 °C for 10 min. The supernatant was neutralized with 1 m Tris-HCl (pH 8.0), which contained chromatin-bound proteins.

### Laser microirradiation and imaging of cells

The accumulation of GFP-fused RNF126 WT and mutants was analyzed as described previously (31). Statistics were calculated by blind manual counting in an unbiased manner.

### DSB repair activity assay

The non-homologous end joining assay was performed as described previously (32). U2OS cells stably expressing reporter plasmid were plated onto 12-well plates. After 24 h, siRNA and expression plasmid DNA (HA-tagged RNF126, truncation mutants, and OTUB1) were transfected at 1–5 μg. 24 h after the first transfection, siRNA, the expression plasmid, and the I-SceI plasmid were transfected. GFP expression was analyzed 48 h after the second transfection by FACS analysis using FACS Calibur (BD Biosciences).

### FokI assay

Co-localization of GFP-fused RNF126 WT with LacI-mCherry-FokI was assessed as described previously (33).

### Statistical analysis

Data were analyzed using GraphPad Prism software (GraphPad Software, San Diego, CA), and the significance of differences between experimental groups was determined using Student's *t* test. A *p* value of less than 0.05 was considered to indicate a statistically significant result; individual *p* values are denoted by asterisks in the figures.

## Author contributions

H.K., K.M., and Y.K. were responsible for the experimental design, data interpretation, and writing of the manuscript. N.S.L., H.R.C., S.K., J.-H.J., J.L., H.J.L., Y.S., M.K., and J.S.H. conducted most of the biochemical experiments.

## Supplementary Material

Supporting Information

## Footnotes

4 The abbreviations used are:

IR: ionizing radiation
iRIF: ionizing radiation–induced focus/foci
DDR: DNA damage response
ATM: ataxia telangiectasia-mutated
ATR: ATM and Rad3-related
NHEJ: non-homologous end joining
DSB: double-strand break
RING: really interesting new gene
ZF: zinc finger
SFB: S-tag, FLAG epitope tag, and streptavidin-binding peptide tag
IP: immunoprecipitation.
