# Supplementary material for: Ring finger protein 126 (RNF126) suppresses ionizing radiation–induced p53-binding protein 1 (53BP1) focus formation
Source: J Biol Chem. 2017 Nov 22;293(2):588–98. doi: 10.1074/jbc.M116.765602 (PMC5767864; doi:10.1074/jbc.M116.765602)
Supplement: Supporting Information [file supp_293_2_588__index.html]

Ring finger protein 126 (RNF126) suppresses ionizing radiation-induced p53-binding protein 1 (53BP1) foci formation — Ring finger protein 126 (RNF126) suppresses ionizing radiation–induced p53-binding protein 1 (53BP1) focus formation — RNF126 negatively regulates DNA damage response — Supporting Information 

# Ring finger protein 126 (RNF126) suppresses ionizing radiation–induced p53-binding protein 1 (53BP1) focus formation

## Supporting Information

- Supplemental Figures (.docx, 26.2 MB) - 4th revised Supplemental Figures
