# Supplementary material for: Ring finger protein 126 (RNF126) suppresses ionizing radiation–induced p53-binding protein 1 (53BP1) focus formation
Source: J Biol Chem. 2017 Nov 22;293(2):588–98. doi: 10.1074/jbc.M116.765602 (PMC5767864; doi:10.1074/jbc.M116.765602)
Supplement: Supporting Information [file 10.1074_M116.765602_jbc.M116.765602-1.docx]

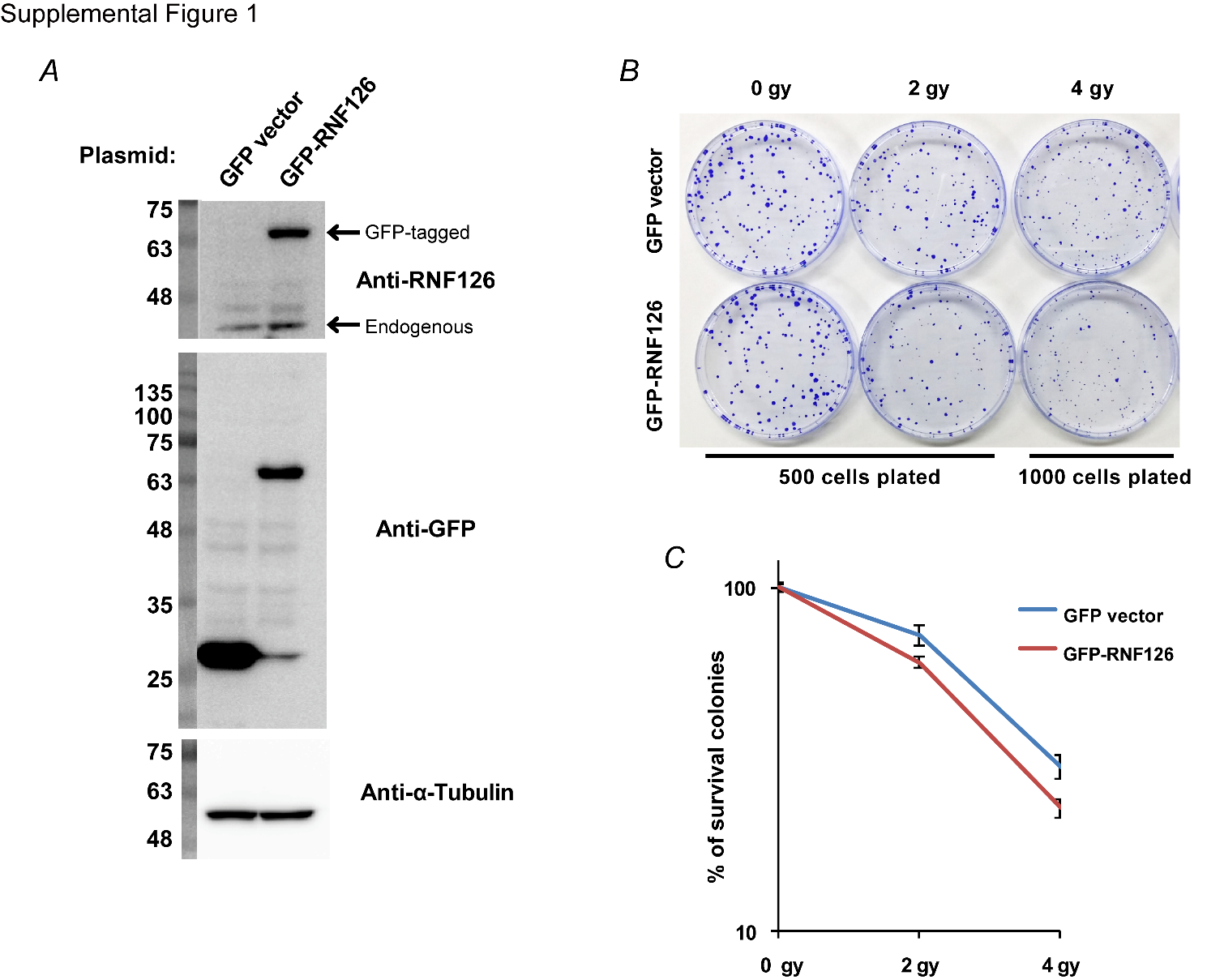


**Supplemental Figure 1. Overexpression of RNF126 affects cell survival to DNA damage.** (A) Overexpression of GFP-tagged empty vector and GFP-tagged RNF126 confirmed by Western blot using anti-GFP, anti-RNF126 and anti-α-Tubulin antibody. (B) and (C) Different doses of ionizing radiation was treated to HeLa cells after cells were transfected with GFP-tagged empty vector or GFP-tagged RNF126. Colonies were stained and counted. *P<0.05.


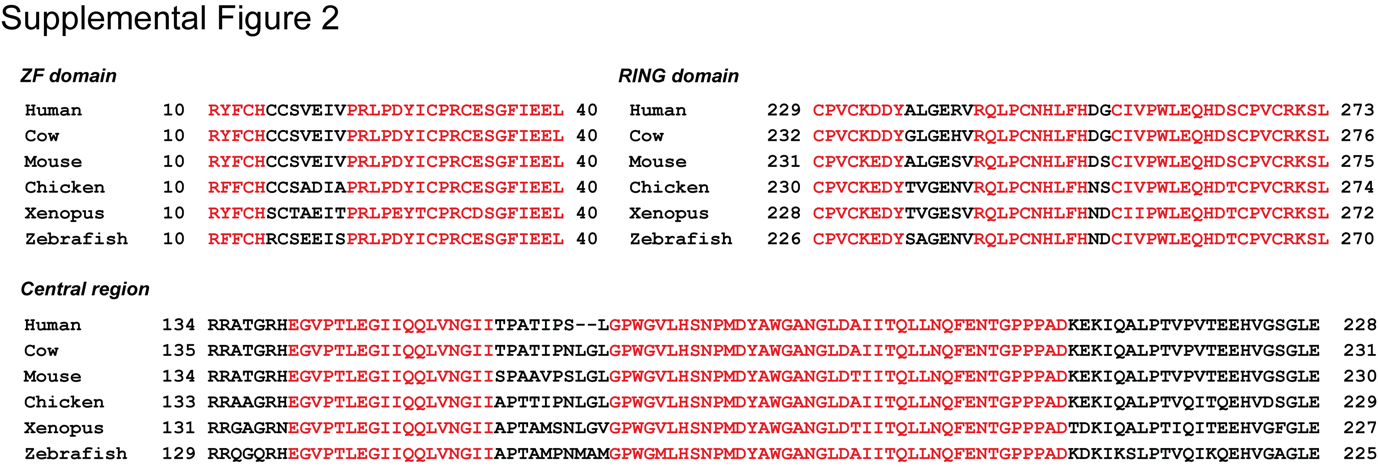


**Supplemental Figure 2. The sequence alignments of the RNF126 ZF, central region and RING domains in mammalian species.** The numbers indicate the region of RNF126 amino acids in each mammalian species.


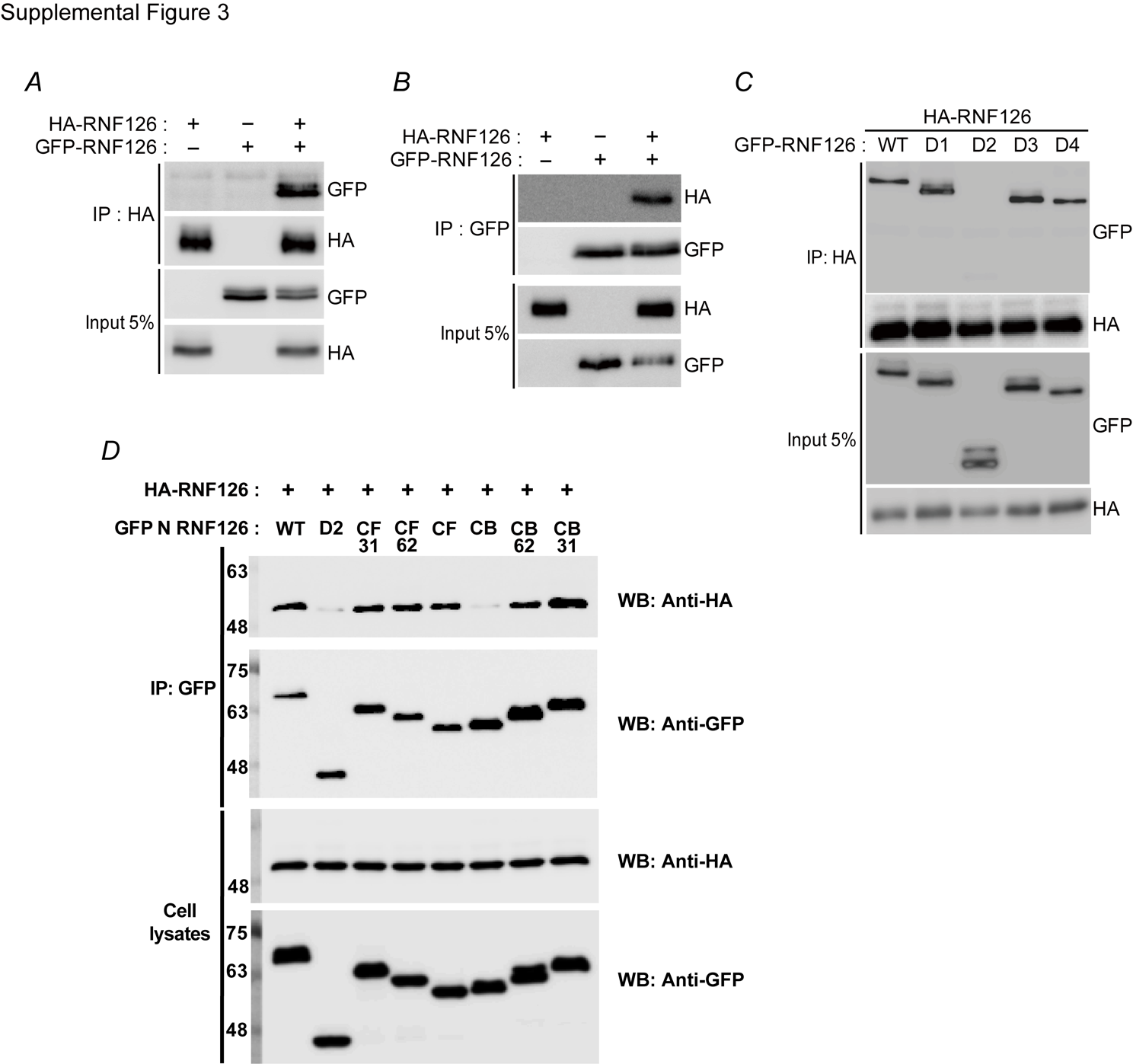


**Supplemental Figure 3. RNF126 forms homo-dimer via its central region.**  (A and B) HA-RNF126 binds to GFP-RNF126 in HEK 293T cells. Twenty-four hours after the transfection with the indicated expression plasmids, the lysates from transfected 293T cells were immunoprecipitated with an anti-HA (A) or anti-GFP (B) antibody and subjected to western blotting analysis using the indicated antibodies. (C and D) The central region is sufficient for RNF126 dimer formation.


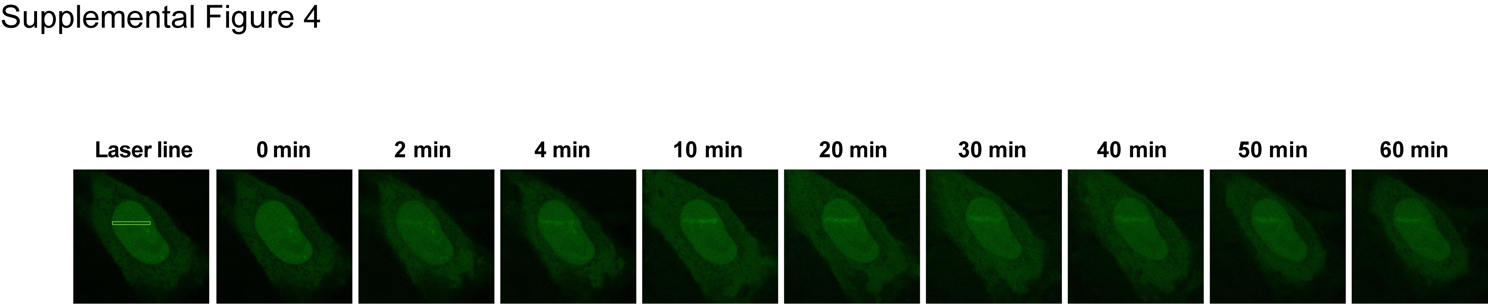


**Supplemental Figure 4. Subcellular translocalization of RNF126 to DNA damage sites.** Kinetics of the translocation of GFP-RNF126 NLS-ZF to DNA damage sites. HeLa cells were transfected with GFP-RNF126 NLS-ZF expression vector. After 24 hrs, transfected cells were treated with BrdU and microirradiated the next day. Immunofluorescent images were taken at time points 0 – 60 minutes.


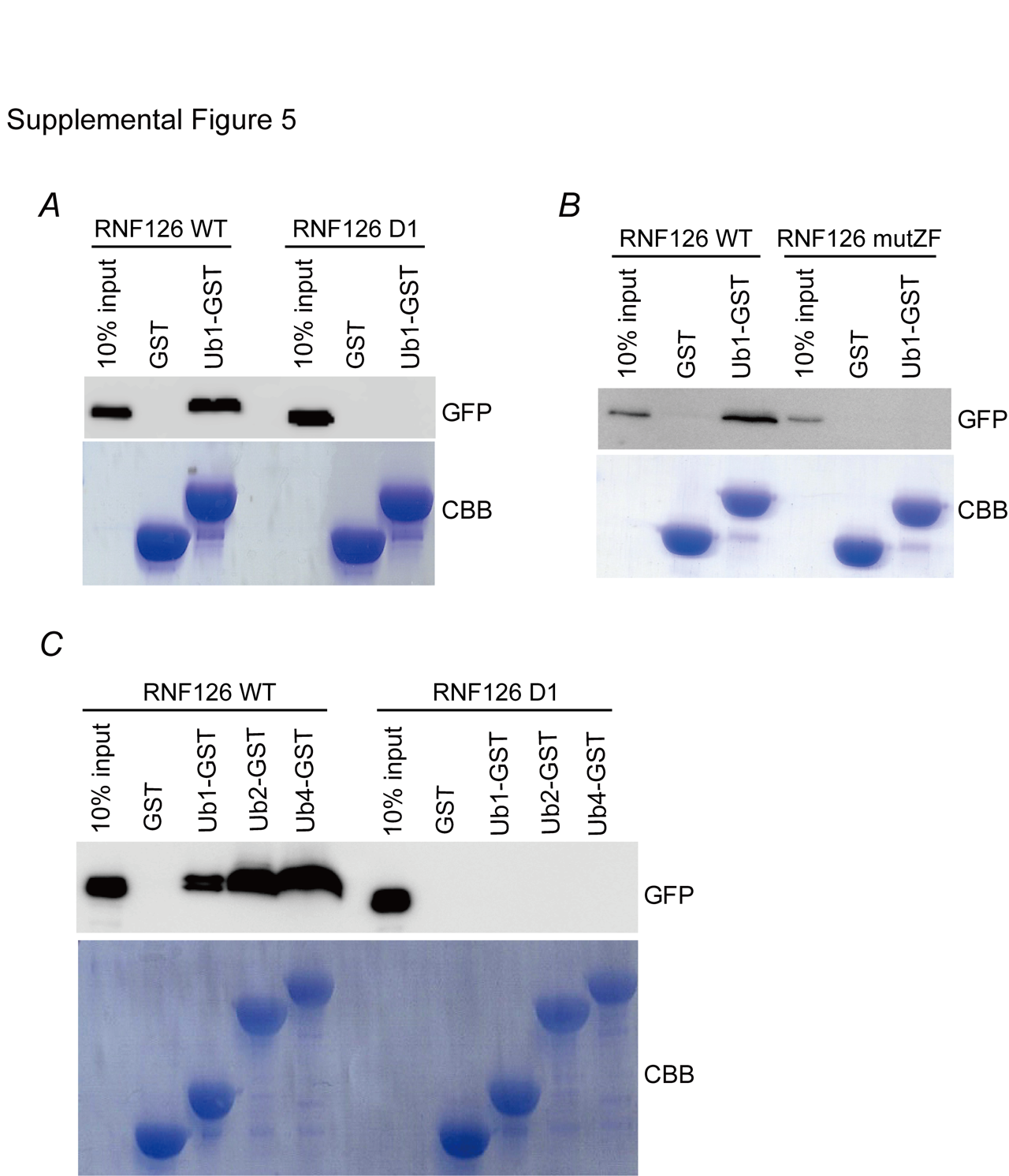


**Supplemental Figure 5. ZF motif of RNF126 binds to ubiquitin.** (A and B) The ZF domain is required for interaction with ubiquitin. GFP-RNF126 WT, deletion or point mutant expression plasmid-transfected 293T cell lysates were incubated with purified GST-Ubi fusion protein. The ubiquitin-associated proteins were analyzed by immunoblotting with the indicated antibodies. Coomassie brilliant blue (CBB) staining was performed to ensure the equal loading of recombinant GST fusion protein. (C) The ZF domain binds better to poly-ubiquitin than mono-ubiquitin.


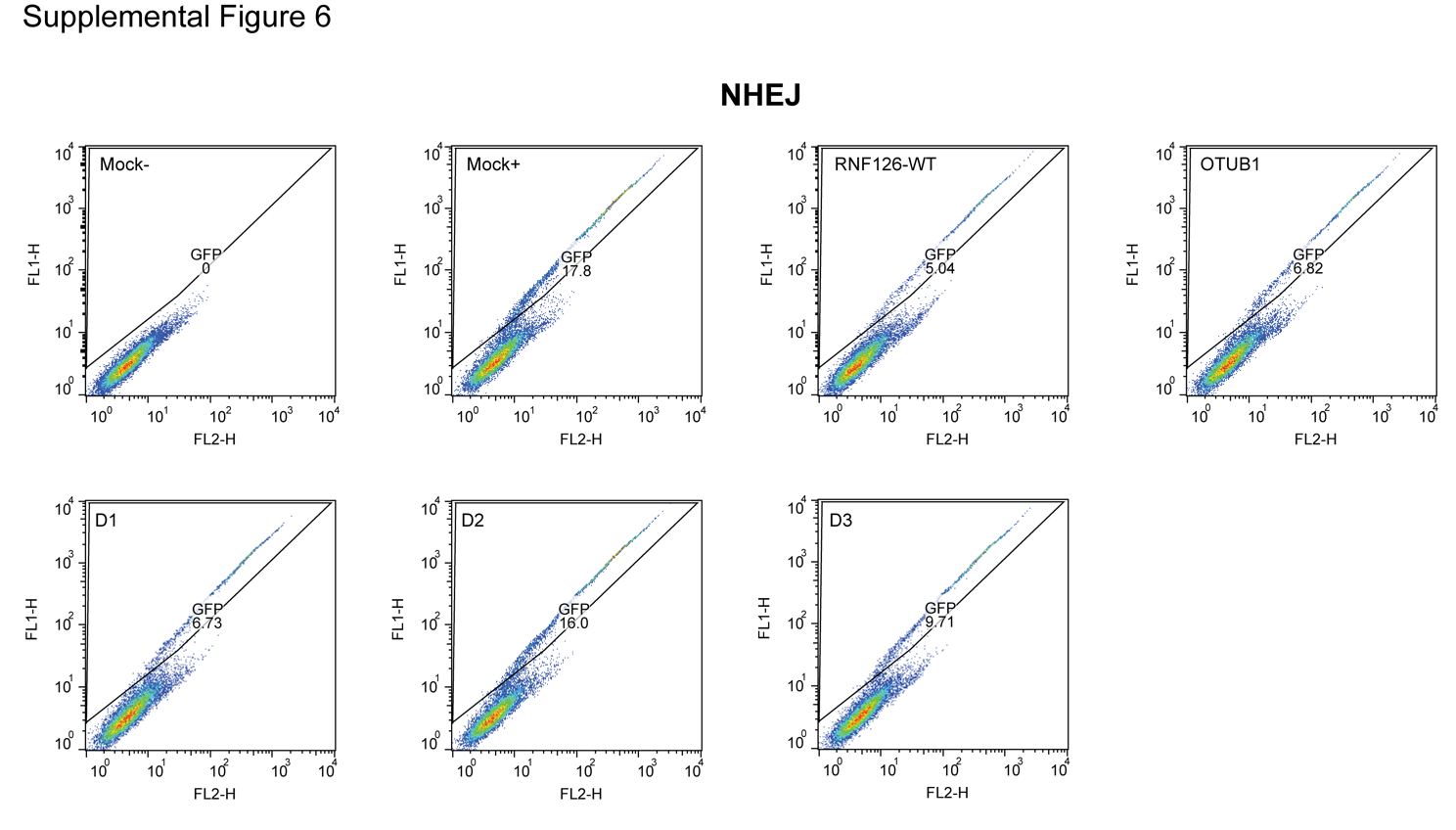


**Supplemental Figure 6. Overexpression of RN126 negatively regulates NHEJ.**

Representative dot plot of NHEJ.
